# A systematic review of outcome and impact of Master’s in health and health care

**DOI:** 10.1186/1472-6920-13-18

**Published:** 2013-02-07

**Authors:** Prisca AC Zwanikken, Marjolein Dieleman, Dulani Samaranayake, Ngozi Akwataghibe, Albert Scherpbier

**Affiliations:** 1 Area Leader Education, Royal Tropical Institute, Amsterdam, The Netherlands; 2 Royal Tropical Institute, Amsterdam, The Netherlands; 3 Department of Community Medicine, Faculty of Medicine, University of Colombo, Colombo, Sri Lanka; 4 ENAULD Health Research and Services, The Hague, The Netherlands; 5 Faculty of Health, Medicine and Life Sciences, Maastricht University, Maastricht, The Netherlands

**Keywords:** Master’s degree programmes, Evaluation, Outcomes, Impact, Systematic review, Public health

## Abstract

**Background:**

The ‘human resources for health’ crisis has highlighted the need for more health (care) professionals and led to an increased interest in health professional education, including master’s degree programmes. The number of these programmes in low- and middle-income countries (LMIC) is increasing, but questions have been raised regarding their relevance, outcome and impact. We conducted a systematic review to evaluate the outcomes and impact of health-related master’s degree programmes.

**Methods:**

We searched the databases Scopus, Pubmed, Embase, CINAHL, ERIC, Psychinfo and Cochrane (1999 - November 2011) and selected websites. All papers describing outcomes and impact of health-related Master programmes were included. Three reviewers, two for each article, extracted data independently. The articles were categorised by type of programme, country, defined outcomes and impact, study methods used and level of evidence, and classified according to outcomes: competencies used in practice, graduates’ career progression and impact on graduates’ workplaces and sector/society.

**Results:**

Of the 33 articles included in the review, most originated from the US and the UK, and only one from a low-income country. The programmes studied were in public health (8), nursing (8), physiotherapy (5), family practice (4) and other topics (8). Outcomes were defined in less than one third of the articles, and impact was not defined at all. Outcomes and impact were measured by self-reported alumni surveys and qualitative methods. Most articles reported that competencies learned during the programme were applied in the workplace and alumni reported career progression or specific job changes. Some articles reported difficulties in using newly gained competencies in the workplace. There was limited evidence of impact on the workplace. Only two articles reported impact on the sector. Most studies described learning approaches, but very few described a mechanism to ensure outcome and impact of the programme.

**Conclusions:**

Evidence suggests that graduates apply newly learned competencies in the field and that they progress in their career. There is a paucity of well-designed studies assessing the outcomes and impact of health-related master’s degree programmes in low- and middle-income countries. Studies of such programmes should consider the context and define outcomes and impact.

## Background

Many publications have addressed the need to train more health workers to meet the human resources for health crisis [1-3] including the shortage of higher cadre staff in public health [3]. Recently, it was questioned whether training of higher level cadres in public health prepared graduates with competencies that are relevant to low- and middle-income countries (LMIC) [4-6], and similarly in high-income countries [7,8]. The question about the relevance of (public) health-related higher education is probably influenced by the trend towards outcome-based education for the health professions [9,10] and by the general debate on the assessment of learning outcomes [11] and the impact of higher education [12-14]. Studies of the impact of master’s degree programmes have mainly focused on the effectiveness of programmes to meet the economic needs of a country and on their contribution to economic productivity in Africa [15-17].

Since the outcomes and impact of master’s degree programmes are also affected by factors occurring after completion of the programme, it is not easy to separate effects directly related to programmes and other influences. Outcomes and impact are thus not easy to measure, and researchers have to decide what variables to measure, what evaluation methods to use, and how to take into consideration the context in which graduates apply their newly learned competencies to achieve the desired outcome and impact.

Kirkpatrick’s evaluation framework is used in many studies evaluating educational results [18]. It distinguishes four levels of evaluation: reaction (a measure of satisfaction); learning (increased knowledge and skills); behaviour (a measure of behaviour change); and results (a measure of results). Hammick et al. [19] elaborated on Kirkpatrick’s framework by developing four interprofessional outcomes: reaction; modification of perceptions and attitudes, including acquisition of knowledge and skills; behavioural change; and change in organisational practice as well as benefits to clients/patients. In 2010, Rothem et al. [14] developed a logical pathway and benefit chain that identifies improved capacity, improved services and improved outcomes for clients.

**Figure 1:**
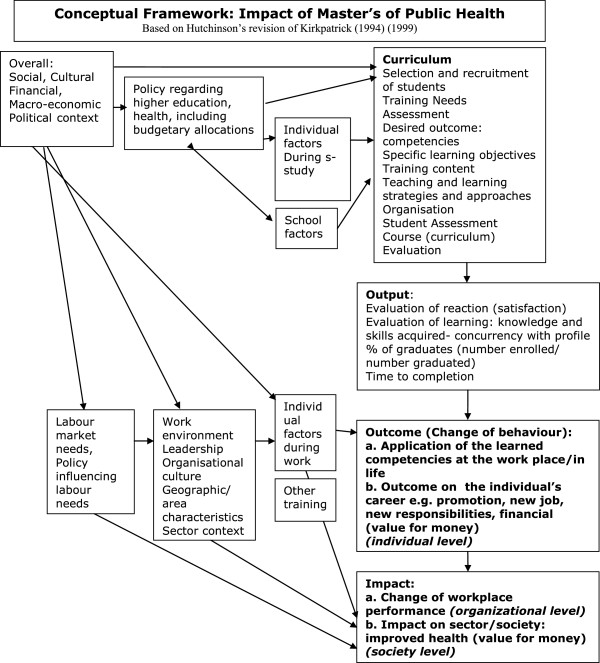
Conceptual Framework: Impact of Master’s Degree Programmes in Public Health.

In this study we used a conceptual framework (Figure 1) based on a revised version of Kirkpatrick’s original framework by Hutchinson [20]. We developed the framework using an iterative process based on the literature review and discussions in the research team. Curriculum output is influenced by the components of the curriculum, the learning objectives, curriculum content and factors such as the selection of students. The learning of students is influenced by individual student and school factors. The curriculum and the learning of students are influenced by higher education policies and budgets.

In the framework, output is defined as the level of satisfaction with the programme expressed by the students and the number of students passing tests, thereby showing they have acquired specific knowledge and skills. The output is not the focus of this study.

In this paper, outcome is defined as the application in practice of competencies learned, such as developing and managing programmes and performing research, and as the effects on careers, i.e. job promotion. Impact is defined as the impact on the workplace, such as changes made by graduates, and the impact on the sector and society, such as improved quality of care.

We identified other factors with a negative or positive effect on programme outcomes or impact, such as individual factors like additional training, personal issues and motivation; work-related factors, such as organisational culture, gender barriers, and income as well as influences from the labour market and overall policies. This paper aims to critically review the methods used to evaluate outcome and impact of master’s degree programmes in the field of health and health care as well as the outcome and impact on the performance of both graduates’ and their workplace.

## Methods

We conducted a systematic review of the literature.

### Search strategy

For the literature search we used the key words: (TITLE-ABS-KEY({master degree} OR {masters degree} OR {masters education} OR {master’s} OR {masters degree in public health}) OR TITLE-ABS-KEY({master degree in public health} OR {masters of public health} OR {masters in public health} OR {master of public health} OR {master in public health}) AND ({impact*} OR {effect*} OR {result*} OR {outcome*} OR {evaluation*} OR {organizational performance*} OR {career mobility})). We searched literature published between 1999 and 30 November 2011, because 1999 was the year in which the Bologna declaration on Master’s educational programmes in Europe was signed [21]. The document types searched for were: (systematic) reviews, primary research studies, evaluation reports and all types of review articles. At the start of the search no limits were set as regards language of publication.

**Figure 2:**
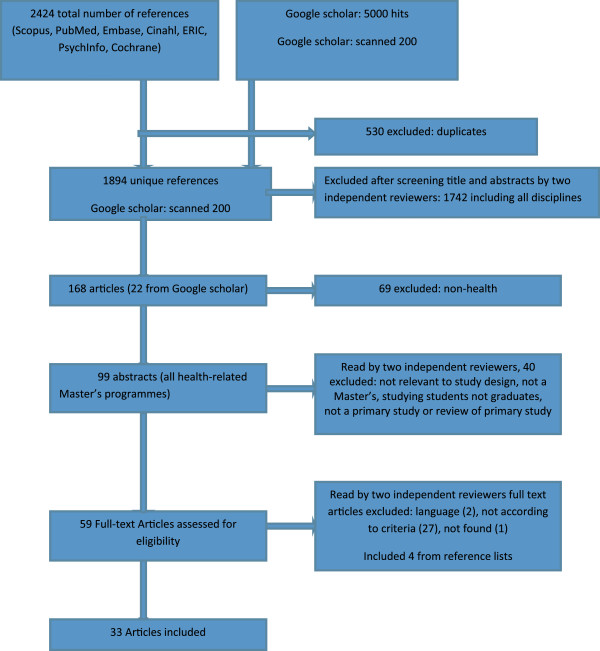
Flow chart of included studies on outcome and impact of health and healthcare-related Master’s.

Title/abstract/keywords were searched in the following databases: Scopus, Pubmed, Embase, CINAHL, ERIC, Psychinfo and Cochrane, as well as Google and Google Scholar by two authors and an information specialist. Figure 2 presents a flow chart of the search. Scanning Google scholar using the same key words yielded about 5000 hits. After excluding duplicates the titles of 1894 unique references were screened by two independent reviewers, which resulted in 168 abstracts. At this stage, we decided to exclude studies of programmes that were not directly related to health or health care. This reduced the number of abstracts to 99 which each were read independently by two of the three reviewers (PZ, DS and NA). After exclusion of abstracts that did not report a primary study or a review of primary studies and had no relevance to the study question, a total of 59 abstracts remained. Of these, two were excluded because of the language (Portuguese). After the reviewers had read the full text of the remaining articles, 29 articles were excluded. Of the thirty articles left, the full text of one could not be retrieved. A further search of the references of the articles with full text revealed additional four relevant articles.

#### Data processing and analysis

The 33 articles were read independently by two researchers in pairs (PZ and NA or PZ and DS). Using the framework developed by the research team, the researchers extracted the following data: name of the programme, target group, programme content/educational methods/assessment methods, time at which graduates were approached (x years after obtaining the degree of interest), level of evidence, study design, methods used to measure outcome and impact, definition of outcome and impact, outcomes studied (application of competences in the workplace, effect on individual careers), working environment of graduates, impact on the workplace, impact on the sector, mechanisms to ensure outcome and impact, the context in which the programme was successful (Additional file 1). The first five articles were analysed by the three researchers together to reach consensus on the data extraction. Whenever there was doubt about data extraction, a third researcher was consulted and consensus was reached through discussion.

The results were synthesised using simple calculation and qualitative analysis. No statistical analysis was performed because of the wide variety of study designs and methods.

## Results

We first present an overview of the studies according to the type and provenance of the programme. Next, we describe how outcome and impact were defined, the methods used to measure these and the level of evidence provided (Table 1). The aggregated results with respect to outcomes (application of competencies in the workplace and career progression) and the impact in the workplace and in society are presented. We then describe the intervention logic and the context of the programmes, the target groups, the contents, the learning approaches and mechanisms to ensure the achievement of outcome and impact.

**Table 1:** Characteristics of the 33 studies reviewed


**Country**	USA (15)
	UK (13)
	Australia, Canada, Ireland (1 each)
	Vietnam (1)
	Systematic review (1)
**Type of Master’s**	Public Health (8)
	Nursing (8)
	Physiotherapy (5)
	General or family medicine (4)
	Occupational therapy (2)
	Others (6: physician assistants, allied health professionals, health communication, pharmacists, global health, psychiatric rehabilitation)
**Level of evidence: all level 4**	‘Triangulation’ design (18) (17 alumni surveys and 1 employer survey)
	Comparison with non-independent reference standard (3, alumni surveys)
	Sequential design (1)
	Mixed methods approach (3): sequential exploratory (2), triangulation (1)
	Qualitative (7*)*
	Systematic review (1)
**Study design**	Quantitative (22)
	Qualitative (7)
	Mixed methods (3)
	Systematic review (1)

### Country and type of programme

The articles reviewed related to programmes in the USA (15), UK (13), Australia (1), Canada (1) and Ireland (1). There was one systematic review of studies on programmes in several high-income countries. Only one study related to a low- or middle-income country (Vietnam).

The articles related to programmes in public health (8), nursing (8), physiotherapy (5), general or family practice (4), occupational therapy (2) and six other professions (physician assistant, allied health professions, health communication, pharmacists, global health, psychiatric rehabilitation). Three articles specified that they dealt with distance-learning programmes, of which two were e-learning programmes. Two studies addressed international programmes, which were open to students from different countries.

### Defined outcome and impact

Programme outcomes were defined in less than one third of the articles. None of the articles gave a definition of impact. Outcomes were defined in terms of the application of competencies, but references to career improvement were virtually absent. Generic descriptions of outcome were used in some studies, such as increased confidence, commitment to the profession and integration with academic skills [22]. Some studies defined outcome as ‘to become an expert in the profession’ [23] or ‘an expectation of improved leadership, management, supervision and teaching in a specific topic’ [24], with specific skills added [25]. Stark examined the changes in roles [26]. Others clarified that the programme was set up to meet changed needs by training physicians with a population perspective [27,28]. Plugge and Cole [6] reported quite broadly defined learning outcomes, while Calvert and Britten [29] reported learning objectives only.

### Methods used to study outcomes and impact

A total of 22 articles used quantitative methods, of which 21 reported the use of self-reported alumni surveys and 1 used an employer survey. In one article two quantitative methods were combined: an alumni survey with an employer survey. Three articles used a mixed methods approach: alumni survey combined with either focus group discussions, or focus group discussions and in-depth interviews or group interviews with students. Of the seven articles using qualitative methods, six reported the use of one method only: either focus group discussions (2), semi-structured interviews (3) or unstructured individual interviews (1). Only one qualitative study used two methods (semi-structured interviews and focus group discussions). One article was a systematic review (see Table 2).

**Table 2:** Methods used to study outcome and impact


**Quantitative methods (22)**	- Alumni survey (20)
	- Employer survey (1)
	- Alumni survey combined with employer survey (1)
**Mixed methods (3)**	- Alumni survey and focus group discussion
	- Alumni survey and in-depth interview and focus group discussion
	- Alumni survey and group discussion
**Qualitative methods (7)**	One method only (6):
	- focus group discussions (2)
	- semi-structured interviews (3)
	- unstructured interviews (1)
	Two methods (1):
	- semi-structured interviews and focus group discussions
**Systematic review (1)**	

For the studies alumni were approached immediately after graduation [23,24], one year after graduation [30], at least three years after graduation [26,31], and up to thirty years after graduation [32]. Eleven studies did not report how many years after graduation alumni were approached.

As for the application of competencies, almost all evidence was from self-reported alumni surveys. Only two studies surveyed employers [33,34]. No pre- or post-measurements were carried out, colleagues were not surveyed, and no other methods were used (such as observation or document review). The majority of studies did not report whether graduates attributed career advancement to their attendance of the master’s programme. Impact in the workplace or the sector/society relied exclusively on self-reports by alumni.

### Level of evidence

All articles evaluated education at level 4 (i.e. case series) [35]. One article compared graduates of two different programmes, one article compared graduates from three different programmes and one article compared alumni from different cohorts. Since these articles did not use an independent reference standard, they were all classified as level 4.

The quality of the studies was further specified based on the Mixed Methods Appraisal Tool [36]. The design of the articles using alumni surveys only (17) was classified as triangulation, because of the concurrent use of closed and open questions. This classification is questionable, however, in the case of studies that did not use other methods or qualitative results to interpret the quantitative data. A study that used an employer survey with a time series analysis was also classified as triangulation [33]. One study used a sequential design with alumni and employer surveys [34]. Only three articles used a mixed methods approach. Cragg and Andrusyszyn [37] mention ‘four focus groups with a total of nine participants’, which does not meet the quality criteria for focus group discussions [38]. The study designs were generally of low quality [36], ie comprising of case series and depending mostly on self-reporting, with little triangulation.

### Studied outcomes: application of new competencies in the workplace

There is reported evidence that graduates applied at least some of their newly acquired competencies in the workplace. They reported improved leadership skills [30,39,40], better job performance [30,34] or improved skills [34,41]. In the study by Murray [34], employers corroborated employees’ enhanced job skills and job performance as a direct result of the master’s programme. Alumni used their research skills [23,42,43] or were involved in research [22]. In a number of studies, graduates reported improvement in the clinical care they provided [22,42-46] and in their attitude towards patients [43,47]. Alumni also reported enhanced self-confidence [22,23,29,37,43,48,49].

The skills that were reported, were specified in some articles: management [25,30], problem solving [44], use of strategic or new approaches [30,50], academic skills [22], teaching skills [42], presentation skills [51] and a range of public health skills [52]. The application of some specific skills were reported, such as clinical practice [49,50], health education and community approaches [33], pharmacy business skills [25], communicating at a higher level [44], a translation function [37], applying a changed perspective on public health [30] and being better equipped for general practice [53]. The use of generic competencies such as: critical reflection [29,46,48], critical thinking and/or analysis [23,29,46,50], the use of evidence [37,46,47] and critical appraisal of the literature [28] was reported in some articles.

Some articles, however, reported that graduates experienced difficulties using newly gained competencies in the workplace [43]. Mental health nursing graduates reported uncertainty about their role and having to compromise their values. They also experienced a gap between theory and practice [22]. Green [42] reported an increased demand for teaching and expectations of advice.

### Outcomes studied: career

Seven studies identified career improvement as an effect of the programme [27,29,34,44,49-51]. Other studies reported specific job changes, such as a higher position/promotion in the same workplace [24,25,30,39-41,47,54], a new job [25,30,51], increased job responsibilities [40], additional roles [53], a new role [42], a new role at a higher level in the system [41] or an appointment in a position where a Master’s degree was required [37].

Three studies reported graduates pursuing an academic career or an increased involvement in academia [34,42,47]. Others reported more management responsibilities [25,42,46,54], less clinical work [39,42] and more involvement in education [25,40,46,47,53,54]. Some reported monetary rewards, such as a higher salary [24,31,54,55] or a higher grade [43,54]. Two articles specifically reported new affiliations [30] and membership of a professional organisation [34]. In some articles alumni reported increased job satisfaction [34,40,47,50] or a higher level of career satisfaction [49]. A number of alumni reported pursuing other studies [30,40,47,54,56] or a PhD degree [23,40].

### Impact studied: in the workplace

Gijbels et al. in their systematic review [43] reported limited evidence of a direct impact on organisational changes and changes in service delivery, including Brooker’s article on improvement in patients’ and carers’ knowledge. Self-reported retention of General Practitioners was described by Baron et al. [53]. Alumni reported the publication of books or book chapters and conference presentations in the articles by Tsimtsiou et al. [47], Richardson et al. [40] and Schattner et al. [39]. Richardson et al. [40] reported popular publications, such as brochures and educational videos. In addition Schattner et al. [39] reported completed research projects and research grants. Davis et al. [30], Cragg and Andrusyszyn [37] and Perry et al. [44] noted that graduates reported encountering resistance in the workplace when trying to implement changes.

### Impact studied: on sector and society

Only two studies mentioned any impact on the sector or on society. In their systematic review, Gijbels et al. [43] reported limited evidence of benefit to patients and carers. They cited evidence from Brooker, for example on mental health care and improvements in patients’ and carers’ knowledge and shorter hospital stays. Richardson et al. [40] stated that graduates from an online master’s programme in occupational therapy reported launching community programmes, developing hospital and clinic programmes and receiving funds for development grants written by graduates. They were also involved in advocacy for improved client benefits and in state regulatory legislative issues [40].

### Intervention logic and context of health-related master’s degree programmes

We use the framework developed for this study to describe different aspects of curricula and the wider context of programmes and graduates’ work settings to identify if and how studies addressed the intervention logic of the programmes.

Regarding the target group: of the eight programmes in public health described in the articles, five did not specify a target group. One article stated that the target group comprised a mix of nurses, healthcare administrators and health educators [29], and two studies reported third-year medical doctors/students as the target group [27,28]. The target group of the Global Health programme was described as a mix of clinicians and non-clinicians [6]. In other studies, the target group was implied in the professional orientation of the programme. For example, nurses were the target group of the nursing programme. Most articles provided little information about the selection and recruitment of students, training needs assessment, specific content and organisation.

A variety of learning methods were used, such as peer group reflection on practice work combined with personal education plans [53], a portfolio combined with course work [53], course and practice work [51], topical modules such as tobacco (including discipline-specific content) [52], mentoring in clinical practice [45,48], different tracks with electives [34] and one track with electives [6]. A Master’s thesis was often mentioned as a final programme component. The assessment methods used were described in ten of the studies. The ten articles described at least two different methods, and some programmes used more methods and combinations of different methods. Course evaluation consisted mostly in end-of-course evaluation procedures.

Learning approaches define how students are expected to learn. Mechanisms to ensure achievement of outcomes and impact included learning approaches as well as approaches to ensure that graduates can apply what they have learned in the workplace. Most of the studies described learning approaches, such as a learner-centred approach [53], and some studies identified a mechanism to ensure the achievement of outcomes and impact, with participants going through a learning cycle of contemplation, assimilation, conflict and resolution [22]. Most of the studies describing such a mechanism also described the learning approach during the programme. Only one study reported that students were selected and the curriculum adapted to their needs as a mechanism to ensure outcomes and impact, although the study did not describe what happened after the programme or what was done during the programme to enhance its impact for alumni.

As regards programme context, a number of articles referred to national or regional government policy (usually health ministries or departments) [22,26,34,39,41,42,46,51],[52] and the labour market [26-28,34,47,53]. These policies and the labour market influenced the initial development of and the reasons for starting a programme, programme content, financing or the number of graduates.

Graduates’ work settings were described only rarely. Baron et al. [53] described a shortage and early retirement of general practitioners.

## Discussion

Although quite a few of the studies we reviewed measured the outcomes of master’s degree programmes in health-related subjects, few measured programme impact. It should also be noted that though the studies focused largely on graduates’ perspectives, and triangulation of data was rare, the review revealed some general issues in relation to the outcome and impact of programmes.

The studies were limited to programmes in high-income countries, except for one programme in Vietnam. This highlights the dearth of literature on health-related master’s degree programmes in low- and middle-income countries. Despite the large numbers of graduates in public health and nursing, programmes in these areas were the subject of only eight articles each.

Interestingly, very few studies *defined the outcomes and impact* before or at the start of the study. This may be explained by the difficulty of defining outcomes and impact of degree programmes like public health, which cover a broad field and are also highly context dependent. However, for master’s degree programmes in physiotherapy or nursing, the impact might be easier to define, for example by measuring reduced duration of patients’ hospital visits or faster recovery [43]. Insofar as outcomes and impact were defined, they were mostly quite generic. This may be inherent in the nature of higher education, with master’s degrees often being pursued to achieve a ‘higher’ level of thinking, such as critical analysis, problem solving etc. On the other hand, however, efforts have been made in a number of countries to assess the learning outcomes of master’s degree programmes at national level [11]. Davis [9], Harden [10] and Harden et al. [57] argue that defining learning outcomes and therefore overall outcome is important to steer content and approaches to learning. This suggests that well-defined learning objectives may be considered to provide sufficient assurance that graduates will be able to perform competently in the workplace and promote changes in society.

The articles we reviewed studied outcomes and impact for different reasons. Interestingly, almost all articles on programmes in physiotherapy, nursing and general practice discussed the question of the validity of a clinical course taught masters. It was often mentioned that even the universities offering the courses did not consider them valuable because they were not aimed at training researchers or did not lead to a PhD degree. Some of the studies were even designed to refute the assertion that these master’s degree programmes were not worthwhile, or to show that they made explicit contributions to either retention of professionals or the development of evidence-based practice in general.

As for *the application of competencies* in the workplace, graduates reported being able to apply their newly gained competencies, whether they were generic, academic or specific. In terms of career-related outcomes, graduates reported being given more responsibilities, receiving promotions, changing jobs and changing careers (for example going into academia or rising within the academic system). Some studies specifically reported higher financial rewards for graduates. In some studies, graduates attributed career changes to the master’s degree, but in other studies the attribution question was not asked. Graduates gain experience over time, which may offer sufficient explanation for career advancement. As for *changes in the workplace,* many studies referred to publishing in both academic and popular outlets and obtaining grants, but also resistance to change in the workplace. Again, these changes were mostly self-reported by graduates. As for *impact on sector/society,* one article [40] very specifically mentioned advocacy, launching community programmes and getting involved in state regulatory issues. What was observed by Gijbels et al. [43] in their review, namely that very few studies identified impact, appears to be confirmed by our review, with impact being largely neglected in the majority of the studies. As for *factors affecting outcome and impact,* some studies reported that resistance to change in the workplace was part of the leadership or organisational culture [43]. Some studies discussed outcomes and impact in relation to the sector, stating that general practitioners or occupational therapists were more motivated to remain in their job as a result of attendance of a master’s degree programme. Only one study discussed the influence of geographical area: Bradley et al. [55] discovered that those who opted to work in a certain area were more likely to receive a higher salary.

The *intervention logic and context of the programme* received only limited attention in most of the studies. Often some information was provided about the target group, programme content and assessment methods. This information may have been readily available from documents. The educational approaches and methods, however, received scant attention. Information about needs assessment, recruitment and selection of students, course facilitators and the organisation and evaluation of courses was limited, if provided at all. Although most of this information could probably have been obtained through document review, many researchers may have considered it to be outside the scope of their study. Hardly any mention was made of the presence of a mechanism to ensure the achievement of outcomes and impact in the workplace and the sector. A possible explanation for this may be that it is generally felt that once students are graduated they fall outside the responsibility of the institution where they received their education. One method of ensuring the applicability of learned competencies in the workplace might be to deliver a part of the curriculum in the future workplace [45,47,51,53]. Also the *work setting of graduates* was mentioned rarely. Some studies reported graduates encountering resistance to change in the workplace, which limited their ability to apply what they had learned. The lack of interest in the setting in which graduates apply what they have learned may be attributable to the considerable amount of time and effort required to fully understand this aspect of the outcomes and impact of master’s programmes. Very often the wider context in which a programme was developed or delivered was described, such as the national or regional policy of ministries or departments of health or of the labour market. These are important factors to be considered.

*The outcomes and impact* of programmes was mostly studied through alumni surveys. Although such surveys may give a reasonably good insight into the careers of graduates and whether they have found their competencies to be useful in the workplace, alumni surveys are self-reported and therefore prone to bias. All study designs were retrospective, using alumni surveys, focus group discussions and semi-structured interviews. The sample sizes of the surveys ranged from 20 [23] to 478 graduates [32], but mostly did not exceed one hundred participants, with response rates varying between 37% and 90%. The limited sample sizes and low response rates undermine the value of the findings. In some studies a mixed group was approached, such as students undertaking a bachelor’s or master’s degree programme [46], postgraduate and master’s degree students [42] or a mix of medical graduates with only a medical degree, another degree or a degree in public health [28]. In the analysis of these studies, however, no distinction was made between these groups, and consequently any changes could not be attributed to the master’s degree or any other level of achievement. In several studies graduates were approached directly after or in their year of graduation. It seems likely that it may have been difficult for these graduates to identify any career changes, as they might still have been in the phase of applying for new jobs or getting back to work. Most studies used instruments that were not validated. Overall the evidence levels were at level 4 and of relatively low quality.

It is therefore not easy to attribute outcomes and impact of master’s degree programmes to specific factors. Triangulation of information from students, peers and employers or superiors or of information obtained using different methods, such as interviews, surveys or observation was rarely reported. It should be noted that interviews with peers and employers can also introduce bias, due to interviewees giving socially desirable answers but also because graduates change jobs often or are given more responsibilities. Observation seems preferable and may be easier for graduates of programmes in physiotherapy or teaching [58], but would probably be more complicated for programmes in public health.

### Limitations

Although there was no language restriction in our literature search, some languages, Chinese for instance, were in fact excluded from the beginning, and this may have biased the results. Our inability to trace one article may have caused bias as well. In some articles, some results or statements of results were not clearly defined. For example, it was not specified what was meant by ‘increased satisfaction’. Job satisfaction was not included in the framework we developed, and this should probably be added. As we stated earlier, the framework makes a clear distinction between outcomes and impact, but in some of the articles and in reality this distinction may be less clear cut.

The framework we developed was helpful in identifying and distinguishing the outcomes and impact of health-related master’s degree programmes. In some studies, however, outcomes and impact were defined differently, and consequently great care had to be taken in the data extraction. In the framework, a clear distinction was made between outcomes and impact, but in reality this distinction may be blurred and there may be some overlap between these categories.

## Conclusion

The number of studies explicitly describing the outcomes and impact of a health-related master’s degree programmes was limited. Despite the growing attention for improving the quality and quantity of human resources for health in low- and middle-income countries, we found only one study on a programme being offered in such a country. Although it is important to define the outcomes and impact of health-related master’s degree programmes in order to identify their contribution to changes in health care, apart from increasing the number of trained professionals, the studies we found revealed a general lack of interest in and provided scant information about these factors. What information was provided was mostly derived from self-reported alumni surveys, and consequently subject to bias. However, although seemingly desirable, a randomised controlled trial over time would be ethically questionable and very difficult to perform. The fact that both the intervention and the outcome take place in a complex environment seems to call for complexity thinking and complexity theory [59]. Another study design that could provide the insights we are after might be a cohort study with follow-up over time, although there are likely to be time constraints. Carefully designed alumni surveys with well-defined outcomes and impact, using triangulation of information from peers and employers, seem to offer a promising approach as well.

Unfortunately, the studies we reviewed rarely considered contextual factors, even though these factors can be crucial in determining whether graduates are able to apply their newly learned competencies and improve the workplace or the sector. We recommend that studies of the effects of master’s programmes address these contextual factors, as we believe such studies will be able to reveal whether graduates of master’s degree programmes are ‘fit for purpose’. These studies might use a realist review [60,61] to enhance the applicability and usability of results to other master’s programmes.

## Competing interests

PZ is the Programme Director of the Master’s of Public Health at the Royal Tropical Institute, Amsterdam, the Netherlands.

## Authors’ contributions

PZ conceived the article. PZ, MD, DS, NA were all involved in designing the conceptual framework, developing the data analysis matrix and in the selection of articles. PZ, DS and NA analysed the articles. All authors contributed to writing and review. All authors agreed with the final content.

## Authors’ information

Prisca Zwanikken, MD, MScCH is a specialist in public health, human resource development, training and HIV/AIDS. She has extensive experience in developing and organising degree and short courses in public health, including quality assurance as well as module development for e-learning. Currently, Dr Zwanikken is KIT’s Area leader for Education.

Marjolein Dieleman, MA, MPH, PhD is a specialist in human resources for health and senior public health expert, coordinator of the “WHO Collaborating Centre for Research, Training and Development of Human Resources for Health” at the Royal Tropical Institute, Amsterdam.

Dulani Samaranayake, MBBS, MSc, MD is a specialist in community medicine with special interest in Human Resources for Health, Occupational Health and Public Health Education. She is currently attached to the Faculty of Medicine, University of Colombo as a lecturer.

Ngozi Akwataghibe, MD, MPH is an experienced project manager and researcher in human resources for health management research. She is the Executive Director of ENAULD Health Research and Services in The Hague area. She is currently working as a World Bank consultant on a healthworker compensation study in Liberia.

Albert Scherpbier MD, PhD is the dean of the Faculty of Health, Medicine and Life Sciences at Maastricht University. He is also professor of quality improvement in medical education and consultant in many international projects.

## Pre-publication history

The pre-publication history for this paper can be accessed here:

http://www.biomedcentral.com/1472-6920/13/18/prepub

## Supplementary Material

Additional file 1 Summary systematic review outcome and impact Master's in Health and health care. Click here for file
